# Alginate Oligosaccharide Alleviates Severe Acute Pancreatitis in Mice via Suppression of Oxidative Stress, Inflammation and Modulation of Intestinal Epithelial Barrier Integrity

**DOI:** 10.3390/biom16060917

**Published:** 2026-06-20

**Authors:** Xianglong Ou, Yi Dai, Xiangyue Hu, Yuan Liu, Shibin Yuan, Le Wang, Bangyuan Wu, Tingting Fang

**Affiliations:** 1 Key Laboratory of Southwest China Wildlife Resources Conservation (Ministry of Education), China West Normal University, Nanchong 637002, China; 2 Nanchong Key Laboratory of Wildlife Nutrition Ecology and Disease Control, China West Normal University, Nanchong 637002, China; 3 School of Basic Medicine and Forensic Sciences, North Sichuan Medical College, Nanchong 637100, China

**Keywords:** severe acute pancreatitis, alginate oligosaccharide, inflammation, oxidative stress, gut microbiota, intestine

## Abstract

Severe acute pancreatitis (SAP) is a life-threatening inflammatory disorder characterized by high mortality and limited therapeutic options. Alginate oligosaccharide (AOS), a marine-derived bioactive polysaccharide, exhibits prebiotic, anti-inflammatory and antioxidant properties that are effective against various inflammatory diseases. In this study, a mouse model of SAP was established by intraperitoneal injection of cerulein (100 μg/kg) and lipopolysaccharide (5 mg/kg), and the mice were pretreated with AOS (200 mg/kg) by gavage for 4 consecutive weeks to explore the potential protective efficacy and underlying mechanisms. The results shown that AOS attenuated the severity of SAP, as evidenced by reduced serum amylase and lipase levels, as well as alleviated histopathological injury in both pancreatic and ileal tissues. AOS suppressed the overproduction of pro-inflammatory cytokines (IL-1β, IL-6, TNF-α) in serum, pancreas, and ileum at protein or mRNA levels. Moreover, AOS effectively diminished pancreatic and ileal inflammatory infiltration and oxidative stress in SAP mice, accompanied by inhibited the TLR4/MyD88/NF-κB pathway and activated the Nrf2/HO-1 antioxidant axis. Furthermore, AOS restored intestinal barrier integrity, as manifested by upregulated expression of tight junction proteins (claudin-1, occludin, ZO-1), reduced serum diamine oxidase, and decreased bacterial translocation from the gut to the pancreas. It was revealed by 16S rRNA sequencing that AOS ameliorated SAP-induced gut dysbiosis by restoring microbial diversity, normalizing the Firmicutes/Bacteroidetes ratio, enriching beneficial genera (*Lactobacillus*, *Blautia*), and enhancing cecal short-chain fatty acid (acetic, propionic, butyric acid) production. Collectively, our findings demonstrate that AOS exerts comprehensive protective effects against SAP through suppression of inflammatory signaling and oxidative stress, as well as restoring gut homeostasis. These results suggest that AOS may serve as a promising prebiotic-based nutritional strategy for the management of SAP.

## 1. Introduction

Severe acute pancreatitis (SAP) is a multifactorial gastrointestinal emergency with an increasing global incidence, characterized by local pancreatic injury and subsequent systemic inflammatory response [1]. The initial pancreatic injury triggers an inflammatory cascade that drives the release of damage-associated molecular patterns (DAMPs), leading to overproduction of pro-inflammatory cytokines and chemokines [2]. Meanwhile, the massive accumulation of oxygen free radicals during SAP pathogenesis contributes significantly to lipid peroxidation, oxidative protein modification, mitochondrial damage, and cellular apoptosis [3,4]. Accumulating evidence has documented the critical roles of extra-pancreatic organs, particularly the gut, in exerting decisive effects on disease severity and progression of SAP [5,6]. Gut dysbiosis and impaired intestinal barrier integrity, characterized by increased mucosal permeability and bacterial translocation, are critical determinants of the initiation, progression, and prognosis of both acute and chronic pancreatitis [7]. The progression of SAP is influenced by multiple factors, involving a dynamic interplay between genetic predisposition and environmental triggers [8]. Nutritional supplements have been demonstrated to be essential for maintaining gut barrier function and mitigating pancreatic severity [9].

Alginate oligosaccharides (AOS), derived from enzymatic or chemical degradation of alginate polysaccharides, have garnered considerable interest as functional bioactive compounds with multiple physiological protective properties [10]. Due to their high water solubility and low viscosity, AOS exhibit a range of biological activities and therapeutic capacities, including anti-bacterial [11], anti-inflammatory [12], and antioxidant properties [13]. These protective effects are achieved through the inhibition of pro-inflammatory cytokine production, directly eliminating reactive oxygen species, and upregulation of endogenous antioxidant enzymes [14]. Concurrently, AOS have been shown to modulate oxidative stress and pro-inflammatory signaling cascades, such as the Nrf2/HO-1 and TLR4/NF-κB pathways [14,15]. Notably, these signaling pathways are closely associated with the severity of pancreatic and intestinal tissue injury in SAP [16,17].

Given the critical role of intestinal barrier dysfunction in pancreatitis pathogenesis, preserving intestinal barrier integrity has emerged as a primary therapeutic target for SAP intervention [18]. Crucially, AOS exerts typical prebiotic effects and exerts intestinal barrier-protective functions via reducing epithelial apoptosis and enhancing the expression of tight junction proteins [19]. Furthermore, AOS alleviates intestinal inflammatory injury not only by maintaining intestinal epithelial barrier function but also by reversing gut microbial dysbiosis [12]. These effects are attributed to a beneficial modulation of the gut microbiota, characterized by enhanced proliferation of beneficial bacteria (e.g., *Akkermansia*, *Lactobacillaceae*, and *Ruminococcaceae*) alongside suppression of pathogenic bacteria (e.g., *Helicobacter* and *Turicibacter*) [20]. Such microbial compositional shift is critical, given that most beneficial bacteria serve as key producers of short-chain fatty acids (SCFAs), including acetate, propionate, and butyrate [19]. Elevated SCFAs exert multifaceted protective effects, which provide not only crucial fuel for energy homeostasis but also contribute to the maintenance of epithelial health [21]. Although the anti-inflammatory, antioxidant, intestinal barrier protective, and gut microbiota regulatory capacities of AOS have been well established in numerous studies, most of these investigations are limited to conventional inflammatory bowel disease and intestinal injury models. However, it remains largely undefined whether AOS can alleviate pancreatic injury induced by SAP via regulating inflammation responses, oxidative stress and gut microbiota homeostasis.

Based on the above findings, we hypothesize that AOS may possess promising capacity to restore pancreas–gut axis homeostasis, preserve gut barrier integrity, and suppress excessive inflammation and oxidative stress in SAP. Accordingly, this study aimed to elucidate the preventive effect of AOS on the severity of experimental SAP and explore the underlying mechanisms. Collectively, our findings offer innovative mechanistic insights into the protective role of AOS pretreatment against SAP-associated multiorgan damage, with the pancreas–intestinal axis identified as the core pathological cascade driving SAP deterioration. This study also highlights that AOS can be developed as a potential preventive strategy for SAP.

## 2. Materials and Methods

### 2.1. Animal Experiments

A total of twenty-four male C57BL/6 mice (6-weeks-old, 18–20 g) were acquired from Chengdu Dossy Experimental Animals Co., Ltd. (Chengdu, China). After being adaptively fed for one week, the mice were numbered sequentially according to their body weight, and a completely random grouping method was adopted via SPSS 22.0 software random number generation. The mice were randomly assigned to four groups (n = 6) designated as follows: Control (CON), SAP, AOS, and SAP+AOS, with no significant difference in initial body weight among all groups. The treatment regimens for each group were as follows: the CON and SAP groups received oral double-distilled water; the AOS and SAP+AOS groups received oral AOS (200 mg/kg/day). The dosages of AOS used in our study was selected based on previous studies where similar dosages of AOS showed bioactivity in mice [12,15,19]. AOS (average molecular weight < 4000 Da) was supplied by Qingdao He Hai Biotechnology Co., Ltd. (Qingdao, China). Following four weeks of daily pre-treatment, SAP was induced in the SAP and SAP+AOS groups. Mice received ten intraperitoneal (i.p.) injections of 100 µg/kg body weight cerulein, spaced at one-hour intervals. After the last cerulein i.p. injection, lipopolysaccharide (LPS, 5 mg/kg body weight) was injected intraperitoneally. Caerulein and LPS were acquired from MedChem Express (Shanghai, China). Mice in the CON and AOS groups served as controls by receiving i.p. injections of an equivalent volume of sterile saline at parallel time points. Mice were humanely euthanized with 60% CO_2_ using a gradual fill method after SAP was induced.

The mice were kept in an air-conditioned room (22–24 °C) with a 12 h light/dark cycle. All mice had free access to diet and water. The animal experiment was carried out in adherence to the protocols approved by the Animal Ethics Committee of China West Normal University (Approval No.: LLSC0036) and conformed to the Guide for the Care and Use of Laboratory Animals.

### 2.2. Serum Lipase and Amylase Activities

The blood sample was centrifuged at 4 °C for 10 min (3000× *g*) to isolate serum, which was aliquoted and stored at −80 °C before analysis. The lipase and amylase kits (Nanjing Jiancheng Bioengineering Institute, Nanjing, China) were used to assess the serum lipase and amylase activities, following strict accordance with the manufacturer’s instructions. Serum amylase and lipase levels are expressed as U/L.

### 2.3. Measurement of Antioxidant Property Indicators

About 1 cm of tissue was homogenized in saline at a 10% (*w*/*v*) ratio and the homogenate was then centrifuged at 4 °C and 5000× *g* for 15 min to obtain the supernatant. The levels of malondialdehyde (MDA), total superoxide dismutase (T-SOD), myeloperoxidase (MPO), and glutathione (GSH) in serum and supernatant of tissues were evaluated using related kits (Nanjing Jian Cheng Bioengineering Institute, Nanjing, China), following the manufacturer’s instructions.

### 2.4. Measurement of Inflammatory Cytokines

Pancreatic tissues were homogenized with a 10% mass fraction using saline, and the supernatants were collected (4 °C, 5000× *g*, 15 min). The colorimetric method was used to measure the serum and pancreatic concentrations of inflammatory markers of tumor necrosis factor-α (TNF-α), interleukin-1β (IL-1β), and interleukin-6 (IL-6) via commercial immunoassay ELISA kits provided by Jingmei Biotechnology Co., Ltd. (Yancheng, China).

### 2.5. Histological Examination

Following euthanasia, pancreatic and ileal tissues were collected and fixed in 4% paraformaldehyde for histological evaluation. After being fixed in 4% paraformaldehyde for 24 h, the tissues were sequentially rinsed, dehydrated through a graded ethanol series, cleared with xylene, and embedded in paraffin. For histological analysis, 5 µm-thick sections were hematoxylin and eosin (H&E)-stained and observed under a light microscope (Nikon Eclipse C1-L, Tokyo, Japan). The severity of pancreatic lesions and ileal histological damage was assessed based on previously established grading criteria [19,22].

### 2.6. Determination of Indicators of Intestinal Barrier Function

To evaluate the degree of intestinal mucosal injury and alterations in intestinal mucosal permeability, the level of diamine oxidase (DAO) in serum was determined using a commercial kit (Nanjing Jian Cheng Bioengineering Institute, Nanjing, China). Ileal expression of claudin-1, occludin, and ZO-1 was evaluated by immunofluorescence [23]. Briefly, following paraffin embedding, ileum tissues were sectioned using a microtome. The sections were dewaxed in xylene, rehydrated through a graded ethanol series, and subjected to antigen retrieval. They were then incubated overnight at 4 °C with primary antibodies against claudin-1, occludin, and ZO-1. For signal detection, the sections were subsequently treated with matching fluorescent secondary antibodies. Nuclei were counterstained with DAPI, and immunofluorescence images were captured using a fluorescence microscope (Nikon Eclipse C1, Tokyo, Japan).

### 2.7. Fluorescence in Situ Hybridization (FISH) Assays

The FISH assay was applied to detect bacterial translocation with reference to the method described previously [24]. Briefly, pancreatic and ileal sections were subjected to dewaxing in 100% xylene twice for 10 min each and then dehydrated in 100% ethanol. Subsequently, hybridization was performed by incubating the sections with the universal bacterial probe EUB338 (5′-Cy3-GCTGCCTCCCG TAGGAGT-3′), which targets the 16S rRNA gene, at 52 °C for 18 h in a humidified chamber. Following a washing step and DAPI staining, images were captured under a fluorescence microscope (Nikon Eclipse C1, Japan), after which the fluorescence ratio relative to EUB338+/DAPI+ signals was quantified per field of view at ×200 magnification.

### 2.8. Quantitative Real-Time PCR (qRT-PCR)

Total RNA was extracted from ileal tissue using TRIzol reagent (Invitrogen, Waltham, MA, USA), and its integrity was verified. Complementary DNA (cDNA) was synthesized with a PrimeScript RT reagent kit (TaKaRa, Dalian, China). Gene expression of IL-1β, IL-6, TNF-α, and Tubulin was quantified by Quantitative real-time PCR (qRT-PCR) using the primers listed in Table 1. Primers were designed by using NCBI Primer-BLAST and synthesized by Sangon Biotech (Shanghai, China). qRT-PCR was performed using the TB Green™ Premix Ex Taq™ II kit (Takara, Dalian, China) on a QuanStudio 6 Flex Real-Time PCR System (Applied Biosystems, Foster City, CA, USA) to amplify the cDNA samples. The qPCR protocol consisted of an initial denaturation at 95 °C for 30 s, followed by 40 cycles of denaturation at 95 °C for 15 s and annealing/extension at 55 °C for 30 s. The relative mRNA expression levels of the target genes were determined using the 2^−ΔΔCT^ method [25].

### 2.9. Western Blot

Pancreatic and ileal tissues were homogenized in radioimmunoprecipitation assay (RIPA) buffer containing a complete protease inhibitor cocktail (Beyotime Biotechnology, Shanghai, China). After centrifugation (4 °C, 10,000× *g*, 10 min) to remove insoluble debris, the protein concentration in the supernatant was quantified by a BCA kit (Beyotime Biotechnology, Shanghai, China). Proteins were separated by SDS-PAGE and transferred onto PVDF membranes (Merck Millipore Ltd., Carrigtwohill, Ireland). The membranes were blocked with 5% bovine serum albumin (BSA) in TBST and then incubated overnight at 4 °C with primary antibodies against TLR4 (Abcam, Cambrigde, MA, USA), p-p65 (Cell Signalling Technology, Danvers, MA, USA), Myd88 (Abcam, Cambrigde, MA, USA), Nrf2 (Abcam, Cambrigde, MA, USA), Keap1 (Abcam, Cambrigde, MA, USA), HO-1(Abcam, Cambrigde, MA, USA), claudin-1 (Proteintech, Wuhan, China), occludin (Proteintech, Wuhan, China), and ZO-1 (Proteintech, Wuhan, China). After washing, the membranes were incubated with horseradish peroxidase (HRP)-conjugated secondary antibodies. Protein bands were visualized using an enhanced chemiluminescence (ECL) assay kit (Bio-Rad, Shanghai, China). The band intensity was quantified using a ChemiDoc™ XRS+ Imager System (Bio-Rad) and normalized to that of β-actin.

### 2.10. 16S Ribosomal RNA (rRNA) Sequencing

Total genomic DNA was extracted from the fecal samples using the QIAamp Stool Mini Kit (Qiagen, Hilden, Germany). Use a NanoDrop 2000c (Nano DropTechnologies, Wilmington, DE, USA) analyzer to measure the concentration and purity of DNA. The V3-V4 regions of the bacterial 16S rRNA gene were amplified by PCR with primers 338F (5′-ACTCCTACGGGAGGCAGCAG-3′) and 806R (5′-GGACTACHVGGGTWTCTAAT-3′), and the amplicons were sequenced on the Illumina MiSeq platform (Illumina, San Diego, CA, USA). The Quantitative Insights into Microbial Ecology (QIIME, version 1.7.0) was used to demultiplex and denoise the raw sequencing data. Sequences with 97% similarity were assigned to the same amplicon Sequence Variants (ASVs). Alpha and beta diversity, LEfSe (linear discriminant analysis effect size), and redundancy analysis (RDA) and plotting were visualized and statistically analyzed in R (version 2024.12.1).

### 2.11. Short-Chain Fatty Acids (SCFAs) Analysis

The concentrations of SCFAs, including acetate, propionate and butyrate, were measured using a 6890A/5973C gas chromatography–mass spectrometry (GC–MS) system (Agilent Technologies, Santa Clara, CA, USA) as previously described [26]. Briefly, supernatant was collected from fecal samples that had been supplemented with 0.5% phosphoric acid and then mixed with an equal volume of ethyl acetate. Following by mixing with internal standards (4-methylpentanoic acid, Sigma-Aldrich, St. Louis, MO, USA). SCFAs concentrations were determined based on standard curves.

### 2.12. Statistical Analysis

Continuous data are presented as mean ± SEM, and statistical analyses were performed using SPSS 22.0 (IBM Corporation, Armonk, NY, USA). All data were tested for normal distribution via the Shapiro–Wilk test and tested for homogeneity of variance via Levene’s test. Comparisons across groups were conducted using one-way ANOVA followed by Tukey’s post hoc test for parametric data after confirming normal distribution. If the normality assumption was violated, the nonparametric Kruskal–Wallis test on ranks was performed, followed by appropriate post hoc analyses. *p* < 0.05 was considered as a statistically significant difference.

## 3. Results

### 3.1. AOS Supplementation Alleviated Severity of SAP

**Figure 1:**
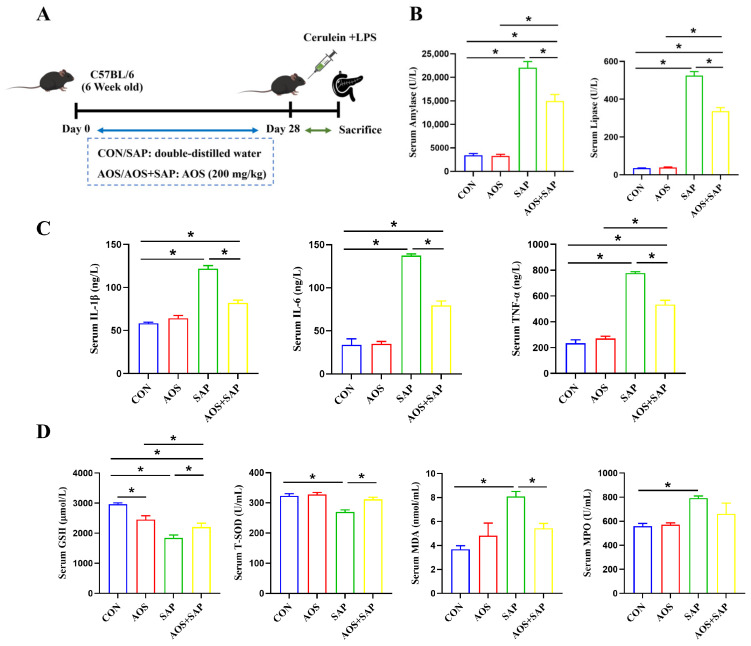
AOS supplementation mitigated the severity of SAP. (**A**) Schematic diagram of mouse groups; (**B**) serum amylase and lipase activities; (**C**) serum IL-1β, IL-6, and TNF-α levels; (**D**) serum GSH, T-SOD, MDA, and MPO levels. Error bars represent mean ± SEM, * *p* < 0.05, *n* = 6.

**Figure 2:**
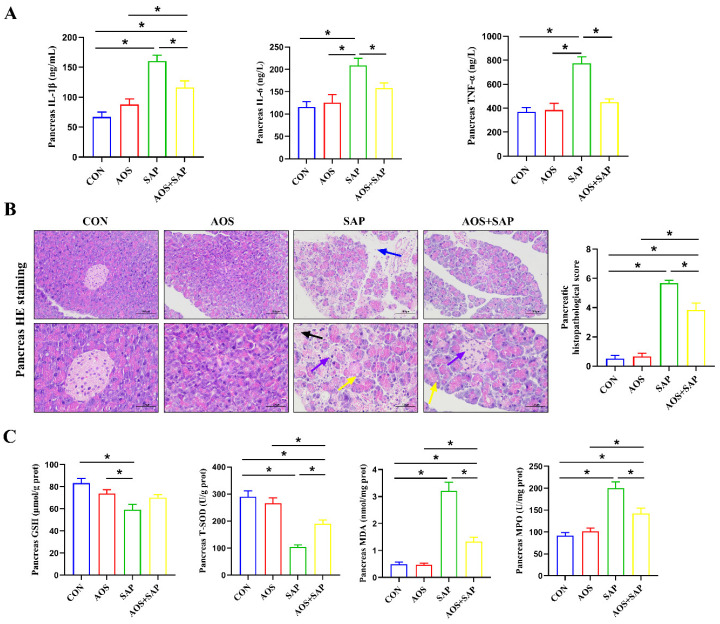
AOS supplementation ameliorated the inflammatory response and oxidative stress in the pancreas. (**A**) Pancreatic levels of IL-1β, IL-6, and TNF-α; (**B**) histopathological changes in the pancreatic sample observed by HE staining (200×); black arrows indicate acinar cell necrosis/vacuolization, yellow arrows indicate acinar cell degeneration, purple arrows indicate inflammatory cell infiltration, and blue arrows indicate scattered inflammatory cell infiltration; (**C**) pancreatic GSH, T-SOD, MDA, and MPO levels. Error bars represent mean ± SEM, * *p* < 0.05, *n* = 6.

To investigate the impact of AOS on SAP, the mice received an oral dose of AOS at 200 mg/kg for four weeks, with intraperitoneal injection of caerulein and LPS after the final AOS administration (Figure 1A). The findings showed that the SAP group of mice exhibited markedly higher pancreatic histopathological scores than the control group (*p* < 0.05), reflected by pancreatic edema, inflammatory cell infiltration, and scattered acinar cell necrosis (Figure 2B). Notably, serum amylase and lipase activities (Figure 1B), which are key biochemical indicators of pancreatitis and whose elevated levels are closely associated with disease severity, were significantly increased in the SAP group relative to the control group (*p* < 0.05). H&E staining confirmed that AOS administration significantly attenuated the pancreatic tissue damage as characterized by reduced pancreatic histopathological score (*p* = 0.002, Figure 2B). Moreover, the elevation of serum amylase and lipase (*p* = 0.038) levels induced by SAP was significantly suppressed by AOS intervention (Figure 1B). Collectively, these data demonstrated that supplementation with AOS substantially alleviated the severity of SAP.

### 3.2. AOS Treatment Protected Mice Against SAP-Induced Inflammatory Infiltration

**Figure 3:**
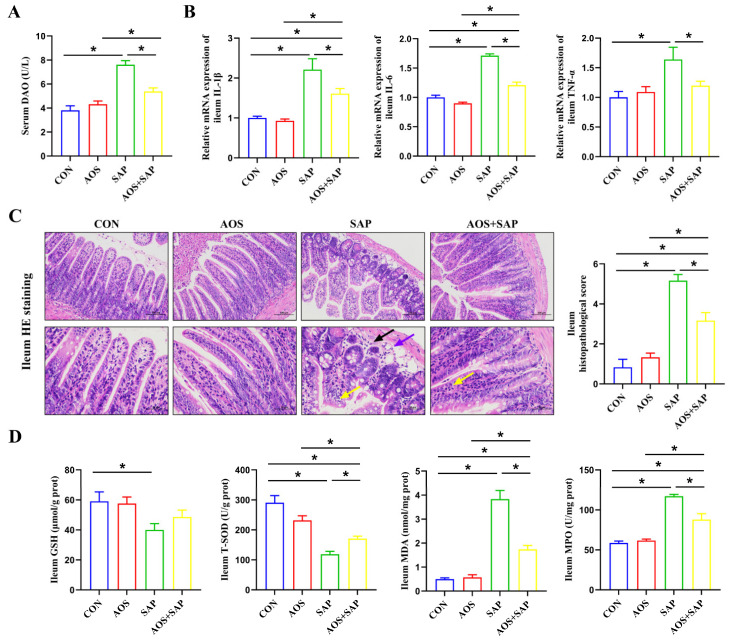
AOS supplementation ameliorated the inflammatory response and oxidative stress in the ileum. (**A**) Level of serum DAO; (**B**) ileal mRNA expression levels of IL-1β, IL-6, and TNF-α; (**C**) histopathological changes in the ileal sample observed by HE staining (200×); black arrows indicate lamina propria edema, yellow arrows indicate epithelial cell hydropic degeneration, and purple arrows indicate lymphocyte and granulocyte cell infiltration; (**D**) ileal GSH, T-SOD, MDA, and MPO levels. Error bars represent mean ± SEM, * *p* < 0.05, *n* = 6.

To further investigate the potential of AOS to mitigate inflammatory damage induced by SAP, the levels of key pro-inflammatory cytokines were measured by ELISA analysis in both serum and pancreatic tissue. It was observed that the levels of IL-1β, IL-6, and TNF-α in serum (Figure 1C) and pancreatic tissue (Figure 2A) were significantly increased following caerulein and LPS induction compared with the control group (*p* < 0.05). Meanwhile, the mRNA expression levels of IL-1β, IL-6, and TNF-α were also enhanced in ileal tissue of the SAP group (Figure 3B, *p* < 0.05). In contrast, this systemic inflammation increase was notably alleviated by AOS supplementation. The AOS+SAP group exhibited markedly lower levels of IL-1β, IL-6, and TNF-α in both serum and pancreatic tissue relative to the SAP group (*p* < 0.05). Moreover, the ileal mRNA expression of IL-1β (*p* = 0.044), IL-6 (*p* < 0.05), and TNF-α was also declined in SAP mice receiving AOS intervention.

### 3.3. AOS Treatment Inhibited Oxidative Stress Caused by SAP

Oxidative-stress-mediated imbalance of the oxidant–antioxidant system is recognized as one of the vital molecular mechanisms and a critical pathogenic pathway in the progression of SAP. In line with this, our findings demonstrated that the SAP group exhibited decreased GSH and T-SOD levels in serum (Figure 1D), pancreatic tissue (Figure 2C) and ileal tissue (Figure 3D) compared with the control group (*p* < 0.05). In contrast, the levels of MDA and MPO in serum (Figure 1D), pancreatic (Figure 2C) and ileal (Figure 3D) tissue were increased in the SAP group (*p* < 0.05). Notably, AOS administration significantly ameliorated this oxidative stress profile in SAP mice. Specifically, AOS treatment restored GSH (*p* = 0.038) and T-SOD (*p* = 0.004) levels in serum and increased T-SOD levels in pancreatic tissue (*p* = 0.007). Moreover, the reduction in the MDA level in serum (*p* = 0.012), as well as reduced MDA and MPO levels in pancreatic and ileal tissue, were also observed in the AOS+SAP group (*p* < 0.05).

### 3.4. AOS Treatment Restored Intestinal Barrier Function and Reduced Bacterial Translocation in SAP

**Figure 4:**
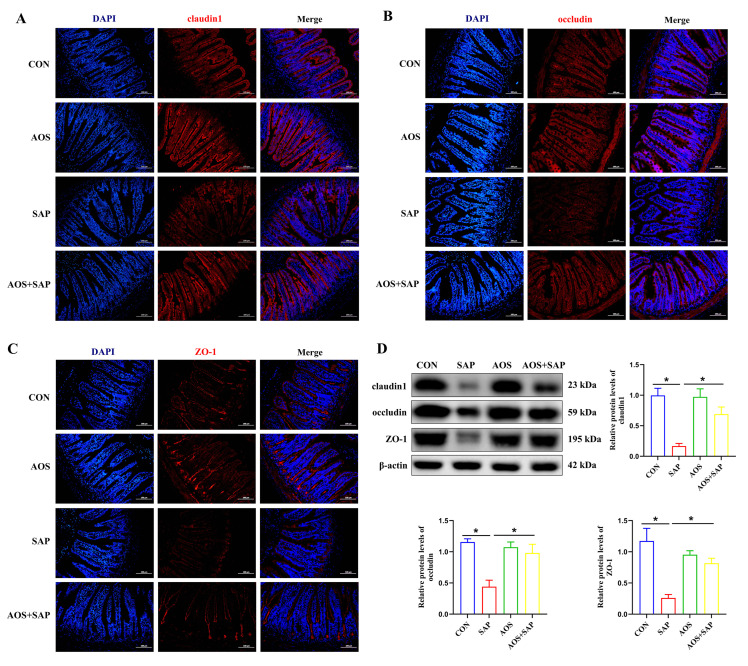
AOS supplementation protected the integrity of the intestinal barrier in SAP. Immunofluorescence analysis for the expression of (**A**) claudin1, (**B**) occludin, (**C**) ZO-1 (200×); (**D**) Western blot analysis of the tight junction protein expression. Error bars represent mean ± SEM, * *p* < 0.05, *n* = 3. Original Western blot images are provided in the Supplementary Materials.

Given the severe ileal and pancreatic injury induced by SAP, we further examined intestinal permeability, intestinal barrier function, and bacterial translocation in this model. As shown in Figure 3A, serum DAO level was significantly elevated in SAP mice compared with healthy controls (*p* < 0.05). Histopathological examination also revealed a markedly higher ileal injury score in the SAP group than in the control group (Figure 3C). Notably, AOS supplementation significantly attenuated these SAP-induced changes in intestinal epithelial permeability and damage (*p* = 0.002). Meanwhile, we assessed the changes in the intestinal barrier function during SAP. As shown in Figure 4, the immunofluorescence staining showed that SAP decreased the fluorescence intensity of claudin1 (Figure 4A), occludin (Figure 4B), and ZO-1 (Figure 4C), consistent with reduced protein expression levels of these tight junction proteins (claudin1, occludin, and ZO-1) in SAP mice (Figure 4D). Notably, AOS intervention effectively promoted the expression of ileal tight junction proteins of claudin1 (*p* = 0.034), occluding (*p* = 0.022), and ZO-1 (*p* = 0.041) in mice with SAP.

**Figure 5:**
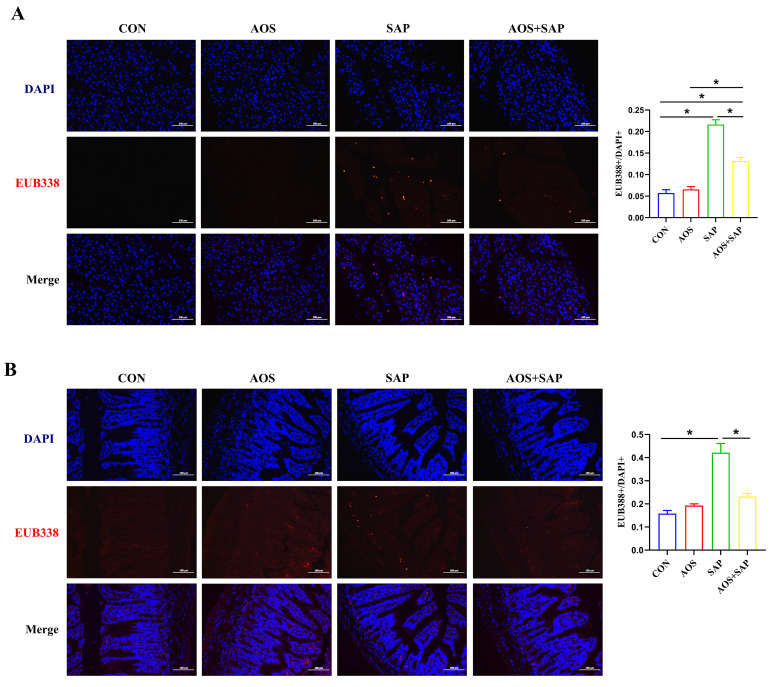
AOS supplementation prevented bacterial translocation in SAP. (**A**) FISH test of the pancreas (200×) and EUB338^+^/DAPI^+^ fluorescence ratio was measured; (**B**) FISH test of ileal epithelium (200×) and EUB338^+^/DAPI^+^ fluorescence ratio was measured. Error bars represent mean ± SEM, * *p* < 0.05, *n* = 6.

Bacterial translocation in the pancreatic and ileal tissue was visualized by FISH assay using the EUB338 probe. The results show that mice with SAP exhibited higher levels of bacterial translocation in both the pancreas (Figure 5A) and ileum (Figure 5B) than the CON group. In contrast, the AOS+SAP group exerted decreased bacterial translocation in the pancreas and ileum when compared with that in the SAP group (*p* < 0.05). Taken together, our data provided evidence that AOS treatment substantially restores intestinal barrier integrity and prevents bacterial translocation from the ileum to the pancreas.

### 3.5. AOS Alleviated SAP by Inhibiting the TLR4/MyD88/NF-κB Signaling and Activating the Nrf2/ HO-1 Signaling Pathway in the Pancreas

**Figure 6:**
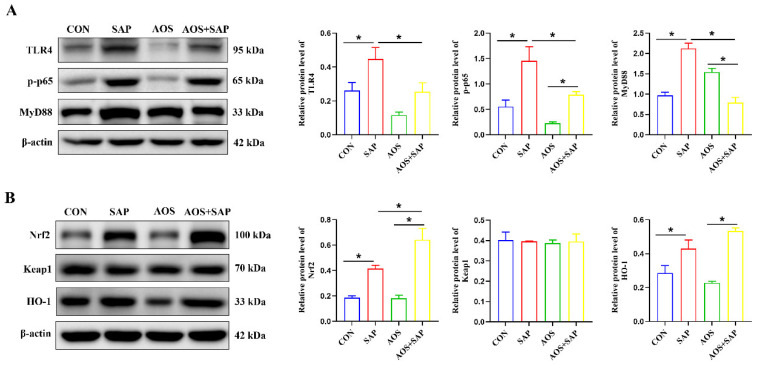
AOS supplementation modulated pancreatic proteins of the TLR4/MyD88/NF-κB pathway and Nrf2/HO-1 pathway in SAP. (**A**) The protein expression levels of TLR4, MyD88, and p-p65 in the pancreas; (**B**) the protein expression levels of Nrf2, Keap1, and HO-1 in the pancreas. Error bars represent mean ± SEM, * *p* < 0.05, *n* = 3. Original Western blot images are provided in the Supplementary Materials.

To elucidate the potential mechanisms underlying the anti-inflammatory effects of AOS in SAP, this study assessed the expression levels of phosphorylate-NF-κB (p-p65) through Western blot analysis to investigate whether AOS exerts an inhibitory effect on NF-κB signaling activation. As shown in Figure 6A, the pancreatic protein expression levels of key pro-inflammatory signaling molecules including TLR4, p-p65, and MyD88 were significantly upregulated in the SAP group compared with the control group. In contrast, AOS treatment reduced the production of inflammatory factors by downregulating the expression of pancreatic key molecules of TLR4 (*p* = 0.049), p-p65 (*p* = 0.035), and MyD88 (*p* = 0.006) in this classical signaling cascade (*p* < 0.05). The Nrf2/HO-1 signaling pathway serves as a pivotal endogenous cellular defensive mechanism, playing a critical function in counteracting oxidative stress and mitigating excessive inflammatory responses under pathological conditions. Western blot analysis revealed that SAP promoted the expression of Nrf2 and HO-1 in the pancreas (Figure 6B). Notably, AOS supplementation further significantly increased the expression of Nrf2 (*p* < 0.05).

### 3.6. AOS Treatment Modulated the Composition of Gut Microbiota in SAP

**Figure 7:**
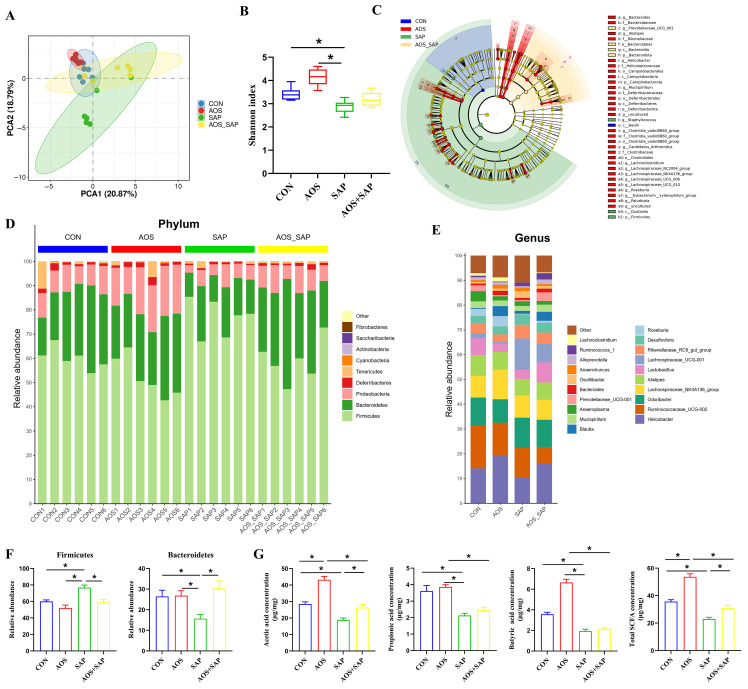
AOS supplementation modulated the gut microbiota composition and SCFAs in SAP. (**A**) The principal component analysis (PCA) based on ASV abundance; (**B**) α-diversity analysis using the Shannon index; (**C**) LEfSe cladogram highlighting biomarker clades enriched in each group; (**D**) composition and differences in the gut microbiota in each group at the phylum level; (**E**) composition and differences in the gut microbiota at the genus level; (**F**) statistical analysis of the relative abundances for Firmicutes and Bacteroidetes in each group; (**G**) the fecal levels of SCFAs including acetic acid, propionic acid, butyric acid and their sum. Error bars represent mean ± SEM, * *p* < 0.05, *n* = 6.

To further elucidate the protective mechanisms of AOS in maintaining intestinal homeostasis and alleviating SAP progression, the regulatory effects of AOS on gut microbiota composition were systematically explored via high-throughput sequencing analysis of bacterial 16S rRNA genes extracted from cecal contents. The principal component analysis (PCA) showed that the gut microbial composition of the SAP group was distinct from that of the CON and AOS groups but was comparable to that of the AOS + SAP group (Figure 7A). Additionally, α-diversity was calculated using the Shannon index to assess the richness and diversity of the gut microbiota. The data indicated that the SAP group exhibited lower microbial richness and diversity compared with the CON and AOS groups (Figure 7B), whereas AOS administration partially restored the gut microbial community disrupted by SAP.

We further examined compositional alterations in the gut microbiota at multiple taxonomic levels using linear discriminant analysis effect size (LEfSe) analysis to identify differentially representative bacterial taxa. The LEfSe results revealed that the structure of the gut microbiota was substantially altered from the phylum to the genus level in both the SAP and AOS+SAP groups (Figure 7C). At the phylum level, Bacteroidetes and Firmicutes were identified as the two predominant phyla (Figure 7D). Following SAP induction, the relative abundance of Firmicutes was significantly elevated, whereas that of Bacteroidetes was reduced (Figure 7F); these changes were notably reversed by AOS supplementation. Furthermore, AOS intervention altered the relative abundance of bacterial community composition at the genus level in SAP exposure mice (Figure 7E). Compared with the SAP group, the AOS + SAP group showed enrichment of *Helicobacter*, *Ruminococcaceae_UCG-002*, *Lactobacillus*, *Blautia*, *Mucispirillum*, *Prevotellaceae_UCG-001*, *Alloprevotella*, *Ruminococcus_1*, along with a decreased relative abundance of *Lachnospiraceae_UCG-001*.

Moreover, to explore the impact of AOS administration on SCFAs generation in the context of SAP-associated gut microbial dysbiosis, we analyzed cecal content samples. The levels of acetic acid, propionic acid, butyric acid, and total SCFA (Figure 7G) were decreased in SAP-induced mice, while these changes could be reversed by AOS intervention.

## 4. Discussion

In this study, we demonstrate for the first time that AOS has a protective effect on pancreatic inflammation via the pancreatic–intestinal axis in a cerulein- and LPS-induced SAP mouse model. AOS supplementation not only enhances anti-inflammatory and antioxidant capacity but also modulates the TLR4/NF-κB and Nrf2/HO-1 signaling pathways in the pancreas. Concurrently, AOS preserves intestinal homeostasis by restoring the epithelial barrier function, reducing intestinal permeability, reshaping the gut microbiota, and promoting SCFAs production, revealing a new microbial metabolic mechanism of AOS against SAP. These improvements in intestinal integrity subsequently minimize bacterial translocation, which in turn attenuates the inflammatory response in both the intestine and the pancreas. Thereby, AOS breaks the vicious cycle of pancreatic–intestinal crosstalk to alleviate SAP, primarily via its antioxidant properties and restoration of intestinal homeostasis.

SAP pathogenesis is initiated by acinar injury and disruption of cell membranes, leading to elevated serum amylase and lipase activities [27], as well as severe histopathological alterations including edema, acinar necrosis, and inflammatory infiltration [28]. This process leads to the release of DAMPs, which in turn promotes the secretion of chemokines and cytokines [4]. An excessive inflammatory response is considered a classical hallmark of SAP in both basic experiments and clinical studies [29]. Accumulating evidence has indicated that AOS exerts cytoprotective roles through its anti-inflammatory properties in a variety of acute and chronic inflammatory disease models [11,19,30]. In our study, we found that AOS pretreatment prevented edema and acinar cell necrosis, reduced serum amylase and lipase activities, and inhibited the production of inflammatory cytokines in the serum and pancreas of SAP mice. Notably, uncontrolled pro-inflammatory cytokine release (e.g., IL-1β, IL-6, TNF-α) is recognized to be a central factor driving damage to extra-pancreatic organs, leading to systemic inflammatory response syndrome (SIRS) and multiple organ dysfunction in SAP [31]. The reduction in digestive enzyme activities and inflammatory mediators observed in this study confirms that AOS exerts a remarkable anti-inflammatory property during SAP.

The intestinal mucosal barrier functions as a selective interface that prevents bacterial invasion while simultaneously maintaining immunological tolerance to commensal flora, which is an organ most susceptible to injury during pancreatitis [22]. Therefore, maintaining the integrity of the intestinal barrier is considered a key factor closely linked to the prognosis of SAP [32]. It is well established that tight junctions, located at the apex of intercellular junctions, regulate paracellular permeability by functionally sealing the paracellular space [33]. Our results demonstrated that AOS pretreatment significantly reduced serum DAO levels and improved ileal histopathological scores in SAP mice. Furthermore, AOS effectively restored the expression and appropriate subcellular localization of tight junction proteins, including claudin-1, occludin, and ZO-1, all of which were severely disrupted following SAP induction. These findings are consistent with previous studies supporting the barrier-protective effects of AOS under diverse pathological conditions [11,15].

Emerging evidence suggests that intestinal barrier dysfunction and bacterial translocation from the gut to the pancreas exacerbate SAP by triggering secondary pancreatic infection and systemic inflammation [34]. The functional improvement in intestinal barrier integrity observed in this study was confirmed by FISH analysis using the EUB338 probe, which demonstrated that AOS pretreatment significantly reduced bacterial translocation from the ileum to the pancreas. Extensive evidence has validated that the TLR4/NF-κB pathway serves as a pivotal mediator of the pro-inflammatory cascade in immune cells following the recognition of bacterial endotoxins derived from translocated bacteria [22,35]. Previous findings show that the anti-inflammatory efficacy of AOS in LPS-induced inflammatory primarily mediated the restriction of the TLR4/MAPK/NF-κB pathway [36]. In this study, we observed that AOS remarkably downregulated the expression of TLR4, p-NF-κB p65, and MyD88. Moreover, our data revealed that AOS significantly reduced the mRNA expression of IL-1β, IL-6, and TNF-α in the ileum, along with a parallel attenuation of pancreatic inflammation. Collectively, our results indicate that AOS alleviates the severity of SAP-associated inflammation, partly by restricting the translocation of gut-derived pathogens, which may potentially suppress the TLR4/MyD88/NF-κB pathway.

During SAP, disrupted pancreatic acinar cells and infiltrated activated inflammatory cells generate excessive oxidants such as reactive oxygen species (ROS) and simultaneously compromise endogenous antioxidant systems, thereby establishing a state of oxidative stress that further amplifies pancreatic tissue injury [37,38]. Previous reports have confirmed the substantial antioxidative capacity of AOS [14], suggesting that AOS may hold protective potential in alleviating pancreatic oxidative stress during SAP. Our study showed that AOS intervention effectively attenuated oxidative stress in mice with SAP, as evidenced by the decreased MDA and MPO levels and restored GSH and SOD activities. Additionally, increasing evidence indicates that oxidative stress-related injury in pancreatitis can be prevented by activating the Nrf2/HO-1 signaling pathway [39]. As the central mediator of the cellular antioxidant defense system, Nrf2 dissociates from Keap1 under oxidative stress and translocates into the nucleus to modulate the downstream antioxidant enzymes such as HO-1 [40]. Accordingly, the Nrf2/HO-1 signaling cascade is widely regarded as the paramount endogenous pathway for counteracting oxidative injury [41]. Consistent with these findings, AOS pretreatment further upregulated the expression of pancreatic Nrf2 and HO-1 in our study. These results reveal a novel molecular mechanism by which AOS enhances cellular antioxidant capacity and attenuates the tissue inflammatory response by activating the Nrf2/HO-1 signaling pathway.

Gut microbiota dysbiosis plays a critical role in the pathogenesis of SAP, not only by modulating innate and adaptive immune responses but also by promoting the disruption of intestinal barrier function, which in turn contributes to pancreatic necrosis and gut–pancreas axis disruption [42,43]. By regulating the proliferation and functional dynamics of the gut microbiota, AOS presents a promising protective potential for ameliorating gastrointestinal disorders and promoting overall host well-being [20]. Based on the relevant literature, we suggest that AOS may alleviate SAP in mice via exerting a profound modulatory effect on the gut microbiota. Similar to the previous study, our findings confirmed disruption of intestinal microbiota homeostasis during SAP [44]. For instance, SAP-induced mice exhibited marked gut dysbiosis, characterized by a decreased abundance of the intestinal flora, a significant expansion of Firmicutes, and a depletion of Bacteroidetes. Notably, these pathological shifts were reversed by AOS intervention. Bacteroidetes and Firmicutes are pivotal for maintaining gut homeostasis; however, an elevated Firmicutes/Bacteroidetes ratio has been closely linked to enhanced intestinal inflammation and systemic inflammatory disorders [45]. Therefore, our results indicate that the anti-inflammatory and barrier-protective effects of AOS are mediated, at least in part, by restoring the compositional balance of the gut microbiota and reversing SAP-induced dysbiosis.

As a well-established probiotic, *Lactobacillus* possesses excellent immunomodulatory and anti-inflammatory functions, which are mediated through the regulation of macrophage polarization and the inhibition of pro-inflammatory cytokine cascades [21]. Also serves as a functional genus with potential probiotic properties, *Blautia* is implicated in the fermentation of dietary fiber and polysaccharides for SCFAs production [46], and its relative abundance is frequently depleted during acute pancreatic [47]. In this study, analysis at the genus level revealed that AOS administration markedly enriched the relative abundance of *Lactobacillus* and *Blautia*, indicating that AOS restored the intestinal microecology not only by restoring overall microbial diversity but also by selectively promoting the proliferation of anti-inflammatory beneficial commensals. In contrast, *Helicobacter*, including both *Helicobacter pylori* and *non-Helicobacter pylori* species, is proven to be a typical pro-inflammatory pathogen that triggers intestinal immune perturbation and aggravates microecological dysregulation [48]. Meanwhile, *Lachnospiraceae_UCG-001* is a key generator of butyrate that is integral to maintaining intestinal health and metabolic homeostasis. Contrary to the previous study [49], the present study revealed that AOS intervention significantly reduced the abundance of the beneficial genus *Lachnospiraceae_UCG-001* while moderately elevating the relative abundance of *Helicobacter*. Such inconsistent microbial alterations reflect disease-specific regulatory characteristics of AOS in modulating complex gut microbial communities across various inflammatory conditions. After AOS intervention, the increase in *Helicobacter* could be an indirect consequence of AOS-driven alteration of the intestinal microenvironment, and its slight enrichment may be an adaptive change during the repair of intestinal microecology. However, the relevant mechanistic investigations regarding the interactive correlation among AOS, *Helicobacter,* and *Lachnospiraceae UCG-001* in pancreatitis are limited; further investigations are required.

Moreover, AOS intervention provided robust protection by preserving the abundance of commensal microbiota and SCFA-generating bacteria such as *Blautia*, *Ruminococcaceae_UCG-002*, *Lactobacillus*, *Prevotellaceae_UCG-001*, *Alloprevotella*, and *Ruminococcus_1* in SAP mice. These bacteria are the crucial symbiotic bacteria in the gastrointestinal tract and are regarded as key participants in maintaining intestinal homeostasis, functions that are closely associated with elevated concentration of beneficial SCFAs including acetic acid, propionic acid, and butyric acid [46,50,51]. As principal metabolites derived from gut microbial fermentation, SCFAs are essential for sustaining the structural and functional integrity of the intestinal barrier. Specifically, SCFAs exert protective effects on intestinal homeostasis via multiple mechanisms, including the attenuation of intestinal inflammation, enhancement of antioxidant capacity, and reinforcement of the intestinal mucosal barrier [52]. AOS are widely acknowledged as prebiotic agents that exert protective functions for intestinal injury by increasing SCFAs production via the selective stimulation of probiotics [53]. Consistent with these prebiotic properties, our study also observed significantly elevated SCFAs levels in SAP mice administrated with AOS. Collectively, these observations strongly suggest that AOS is capable of reshaping the gut microbial ecosystem in SAP mice, creating a favorable microenvironment that promotes the enrichment of SCFAs producing bacteria and enhances endogenous SCFA biosynthesis. Such prebiotic-mediated modulation of the gut microbiota may represent a key mechanism underlying the protective effects of AOS against intestinal barrier dysfunction and systemic inflammation during SAP progression.

Notably, this study has demonstrated that AOS exerts stable preventive effects on SAP-induced pancreatic and intestinal damage, there are still limitations to this research. Only a single dose of AOS, which is derived from previously validated effective doses in analogous intestinal and inflammatory injury models [12,15,19], was used for intervention in the current experiment, and no gradient dose–response verification was performed. Further experiments with multiple gradient doses are required in future studies to clarify the correlation between dosage and preventive efficacy. In addition, our study is limited to male mouse models with a limited sample size per group. Future studies should expand the number of experimental animals, including both male and female, to further explore the role of AOS in SAP, and the clinical validation in humans also needs to be performed before AOS is proposed as an adjuvant treatment strategy for SAP.

## 5. Conclusions

The present study demonstrated that AOS effectively alleviated SAP through a multi-tiered mechanism centered on the gut–pancreas axis. Specifically, AOS supplementation alleviated pancreatic tissue injury, enhanced intestinal barrier function, and mitigated systemic inflammation and oxidative stress. The protective mechanism of AOS was partly attributed to the reconstruction of gut microbial homeostasis and the regulation of the TLR4/MyD88/NF-κB and Nrf2/HO-1 pathways. These results highlight AOS as a promising natural oligosaccharide candidate for the prevention and adjuvant treatment of SAP, while also providing novel mechanistic insights into the crosstalk between the gut microbiota and the gut–pancreas axis in acute pancreatic injury.:

## Figures and Tables

**Table 1:** List of primer sequences.

Gene Name	Primer Sequence (5′-3′)	
IL-1β	Forward	TGACAGACCCCAAAAGATTAAGG
	Reverse	CTCATCTGGACAGCCCAAGTC
IL-6	Forward	CCACCAGGAACGAAAGTCAAC
	Reverse	TTGCGGAGAGAAACTTCATAGCT
TNF-α	Forward	CAGCCGATTTGCCATTTCA
	Reverse	AGGGCTCTTGATGGCAGAGA
Tubulin	Forward	GGCAGTGTTCGTAGACCTGGAA
	Reverse	CTCCTTGCCAATGGTGTAGTGG

## Data Availability

Data will be made available on request.

## Supplementary Materials

The following supporting information can be downloaded at: https://www.mdpi.com/article/10.3390/biom16060917/s1, Original Western blot images for Figure 4 and Figure 6.
