# Supplementary figures and images for: Alginate Oligosaccharide Alleviates Severe Acute Pancreatitis in Mice via Suppression of Oxidative Stress, Inflammation and Modulation of Intestinal Epithelial Barrier Integrity
Source: Biomolecules. 2026 Jun 20;16(6):917. doi: 10.3390/biom16060917 (PMC13297282; doi:10.3390/biom16060917)

Original Western blot images for Figure 4.

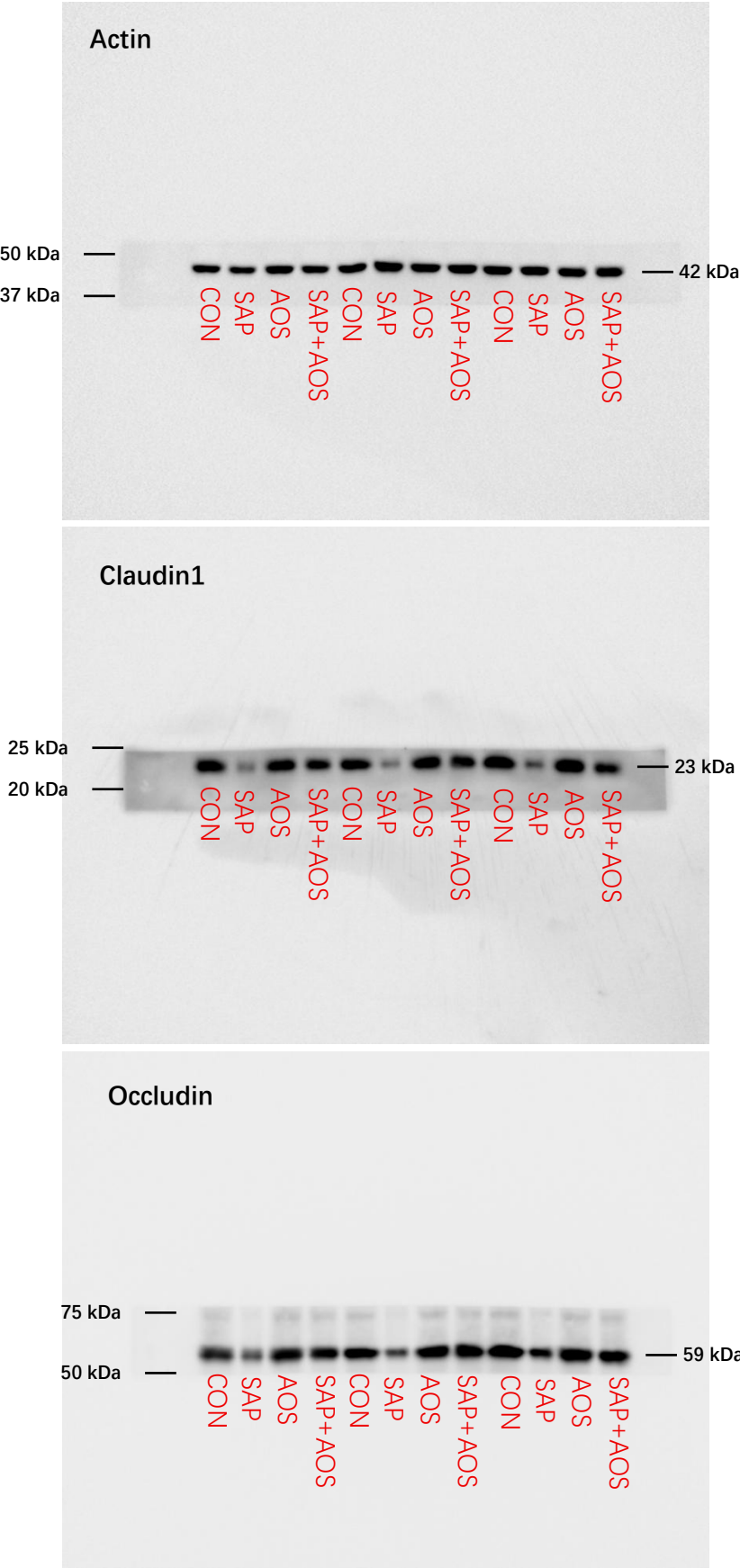

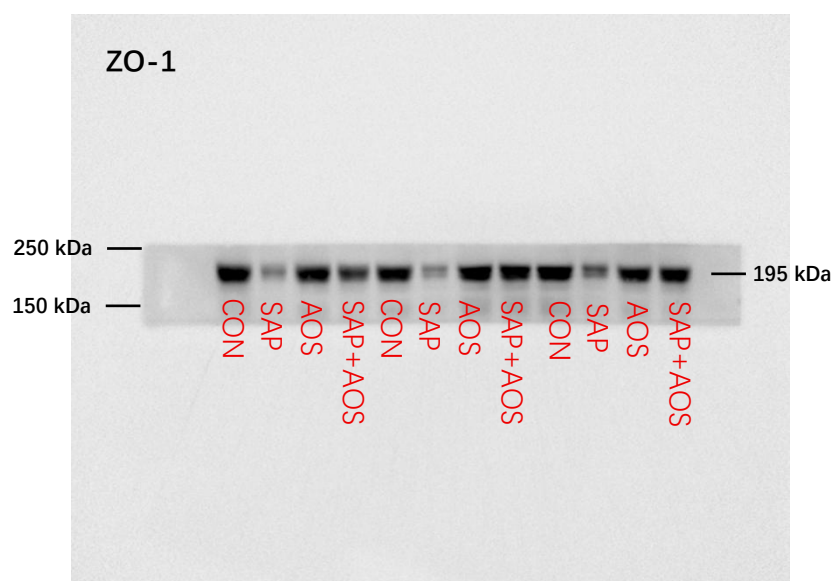

Original Western blot images for Figure 6.

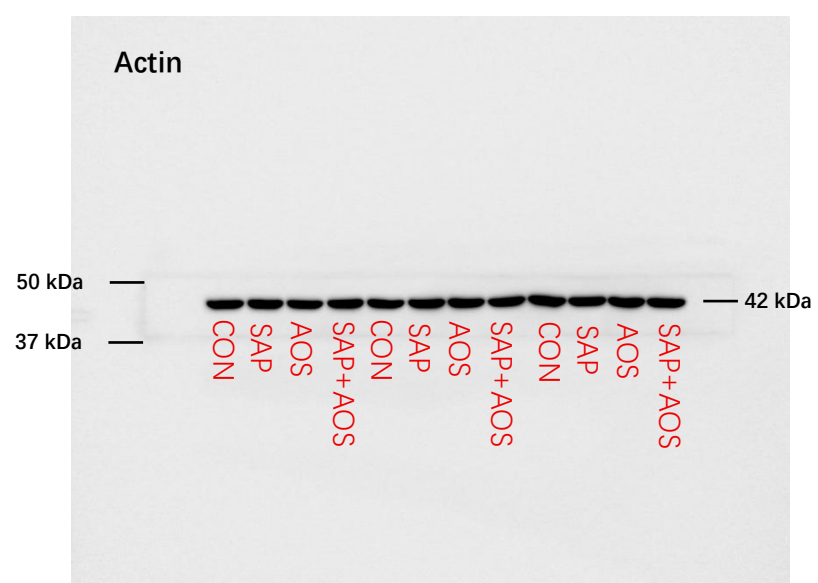

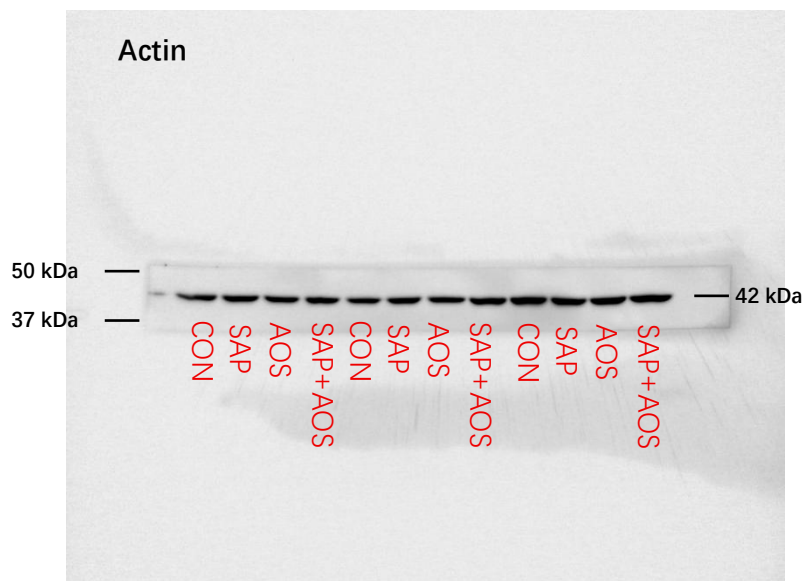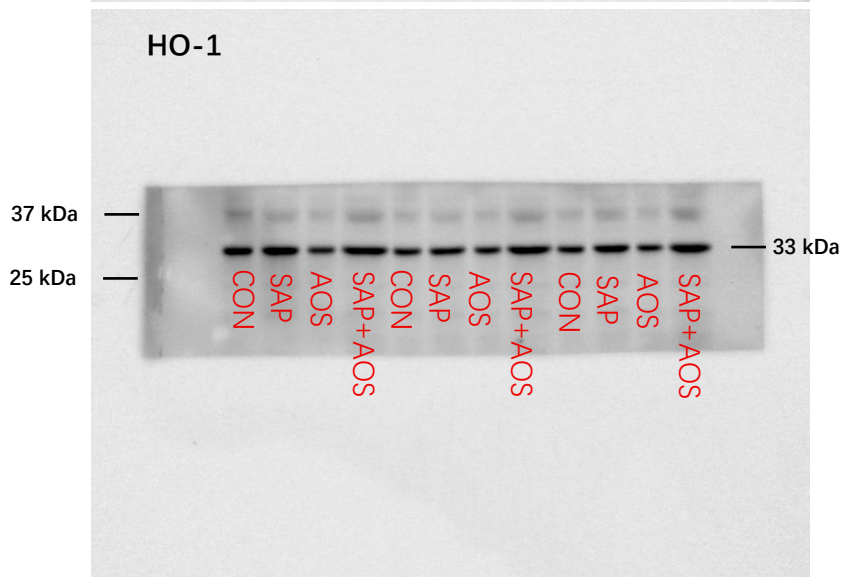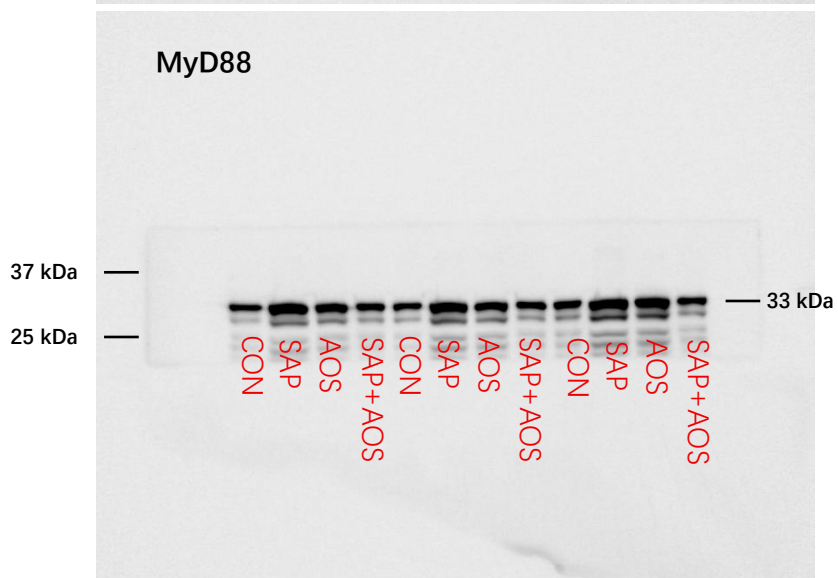

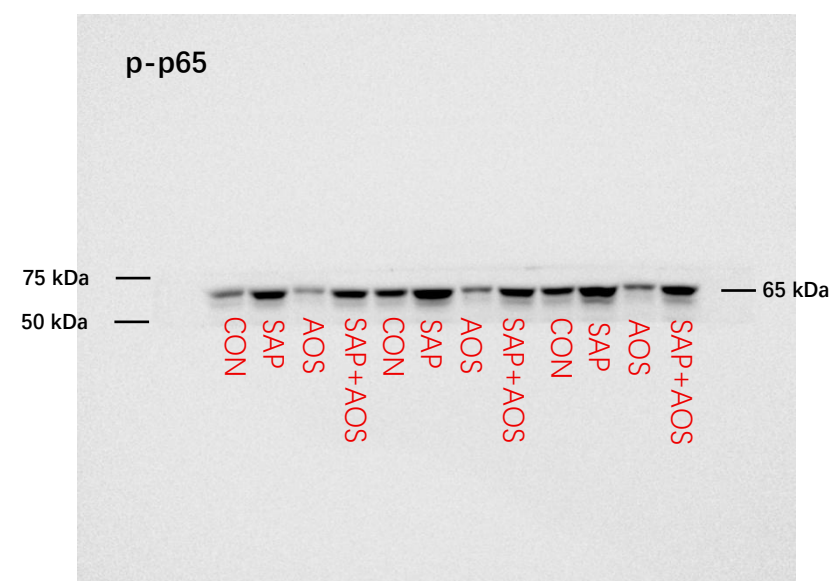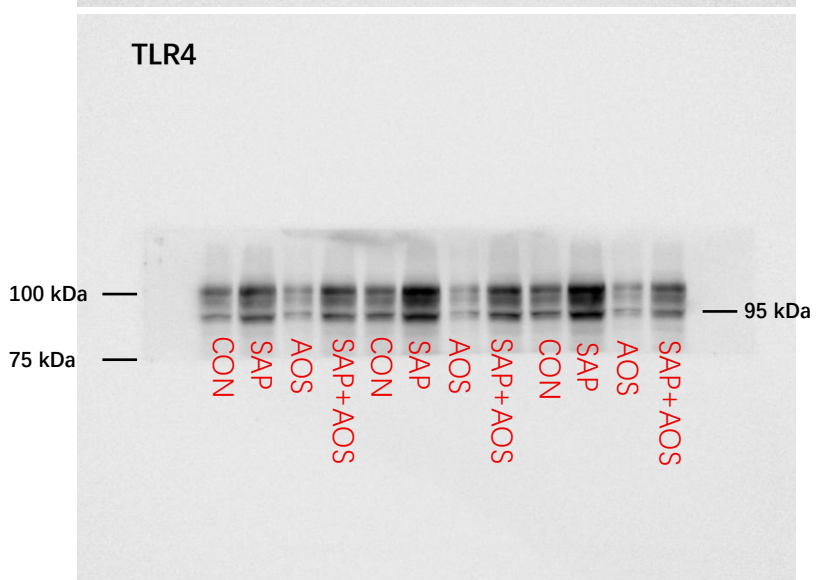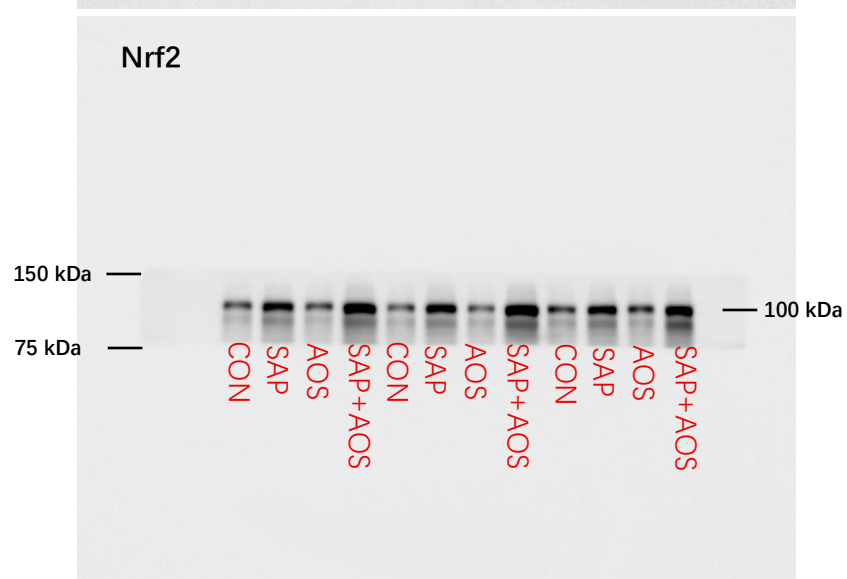

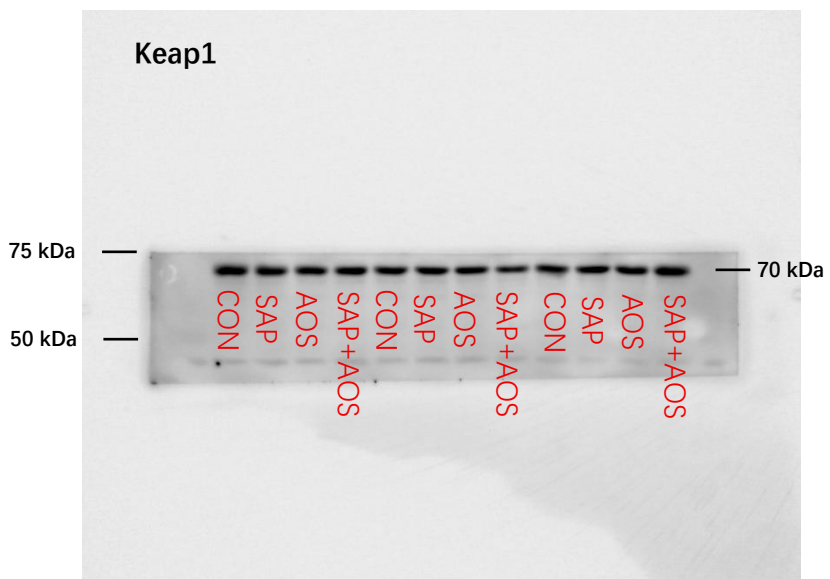

Supplement: Supplementary file 1 [file biomolecules-16-00917-s001.zip › biomolecules-4371733-supplementary.pdf]
